# Call for better response evaluation after neoadjuvant therapy in pancreatic cancer

**DOI:** 10.1093/bjs/znac452

**Published:** 2023-01-12

**Authors:** Claudia Zaharia, Kjetil Søreide

**Affiliations:** Department of Pathology, Stavanger University Hospital, Stavanger, Norway; Gastrointestinal and Translational Research Group, Stavanger University Hospital, Stavanger, Norway; Gastrointestinal and Translational Research Group, Stavanger University Hospital, Stavanger, Norway; Department of Gastrointestinal Surgery, Hepatopancreatobiliary Unit, Stavanger University Hospital, Stavanger, Norway; Department of Clinical medicine, University of Bergen, Bergen, Norway

Pancreatic cancer is believed to be a systemic disease from the very early stages. Hence, although surgical resection provides the best chance of cure, multimodal treatment is essential for long-term survival^1^. Outside the narrow inclusion criteria for clinical trials, up to half of all patients do not recover well enough to start, let alone complete, adjuvant treatment after surgery. This observation has shifted systemic treatment towards a neoadjuvant approach for borderline resectable and, more recently, for upfront resectable disease.

For resectable disease, one of the important intentions of neoadjuvant chemotherapy, in addition to increasing the rate of receipt of systemic therapy, is to avoid a major resection in patients who would develop metastasis early in the course after surgery. The theory is that early metastasis is most likely present at the time of diagnosis, albeit not visible on conventional imaging or by standard tests. Hence, neoadjuvant chemotherapy becomes a test of biology, before proceeding to surgery. For borderline and locally advanced pancreatic cancer, previously considered unresectable, effective yet toxic regimens, such as the combination regimen of fluorouracil, leucovorin, irinotecan, and oxaliplatin (FOLFIRINOX), have made induction therapy an option. Although neoadjuvant therapy for locally advanced pancreatic cancer is arbitrarily being referred to as ‘downstaging’ or ‘downsizing’ by some, it is really a test of tumour responsiveness or biological aggressiveness. Response evaluation by cross-sectional imaging after neoadjuvant treatment is troublesome, and to some degree even unreliable^2^. For one, response evaluation after FOLFIRINOX based on CT shows poor correlation between imaging findings and tissue evaluation at surgical exploration, potentially depriving patients the chance of resection if imaging findings are evaluated for response alone. Predictors of response and robust indicators of resectability are few when based on traditional methods, except for decreased or normalized carbohydrate antigen 19-9 values as a favourable biological sign^3^. Notably, about 10 per cent of the population does not express this glycoprotein marker, so it is not universally useful.

**Fig. 1:**
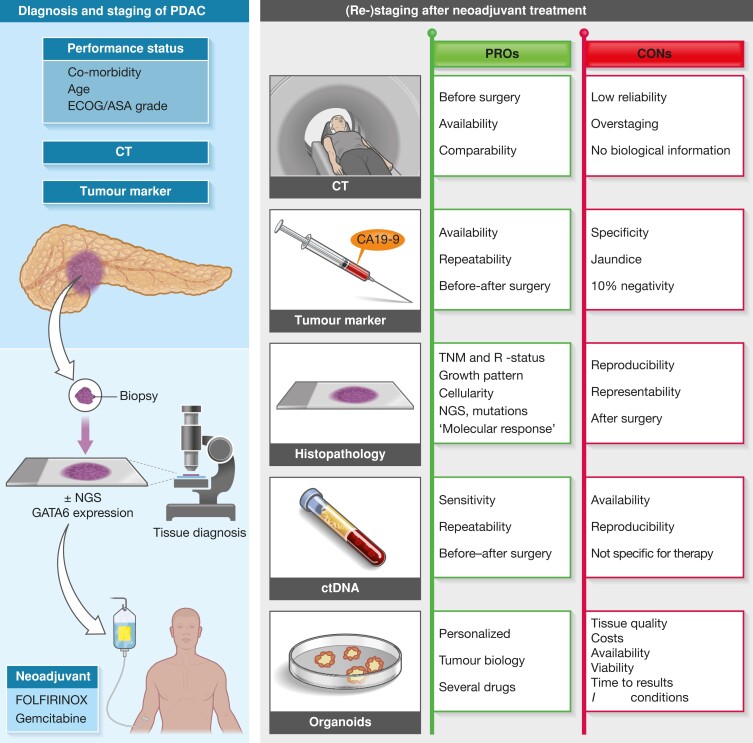
Modes of response evaluation of neoadjuvant therapy in pancreatic cancer **a** Diagnostic and staging pathway before neoadjuvant therapy; **b** conventional and emerging ways of evaluating response, either before surgery or after surgery, with advantages and disadvantages. A combination of methods will likely to be used in the future to best evaluate response at baseline, at interim evaluation, and after completion of therapy. PDAC, pancreatic ductal adenocarcinoma; CA19-9, carbohydrate antigen 19-9; ECOG, Eastern Cooperative Oncology Group performance status; NGS, next-generation sequencing; ctDNA, circulating free tumour DNA.

After pancreatic cancer resection, histopathological examination offers the opportunity to evaluate tumour response to neoadjuvant treatment (*Fig. 1a*). This provides information about the effect of the neoadjuvant chemotherapy by evaluating the viability and characteristics of the cancer cells and remaining tumour bed. On the other hand, tumour response also reflects the sensitivity or resistance of the patient’s cancer cells to a given treatment. For example, very little or lack of evaluated effect on the tumour cells would guide the clinician to change treatment, say from FOLFIRINOX to a gemcitabine-based regimen. However, this would only be a sound approach if the histopathological tumour response evaluation were robust, valid, and reproducible. One of the problems with histopathological tumour response evaluation is the lack of consensus on which scoring system represents best practice; a total of 13 unique scoring systems were found in a recent systematic review^4^. A score that is reliable, valid, and has high accuracy for true tumour regression (or lack thereof) is important, as it will guide the clinicians to continue with same chemotherapy regimen in the adjuvant setting, or indicate whether a change of therapy is warranted.

The number of available scores alone is not the obstacle to agreement—rather the reproducibility and validity of any given score *per se*, it would seem. In a study in *BJS*^5^, several world-leading experts in pancreatic pathology agreed to review neoadjuvant-treated and neoadjuvant-naive specimens using the two most commonly used tumour response scoring systems (College of American Pathologists and MD Anderson)^5^. Even among experts, the correlation was only moderate. One of the issues that might explain interobserver variability is that the evaluation of tumour response is based on subjective criteria which are not specific. One of the challenges identified by the study^5^ was discerning between tumour-induced desmoplasia and treatment-related fibrosis. In addition, the existing scoring systems assess different aspects of treatment effect—some evaluate residual (viable) tumour cells, whereas others focus on regression features in the tumour bed. Differences in number of categories and clear-cut criteria contribute to variation in discriminative prognostic power and ease of application^6^.

A pCR in pancreatic cancer is a rather rare phenomenon. An in-depth study^7^ from Johns Hopkins even suggested that pCR may be a misnomer. Based on data from 39 specimens with a pCR response, they investigated the molecular landscape in tissue and liquid biopsies. A novel concept of tumour regression assessment combining genomic analysis of resected specimens and liquid biopsy data for pancreatic cancer was termed ‘molecular response’^7^. A complete molecular response was associated with a lower risk of recurrence. The study provides a new concept for response evaluation but needs validation as several features could not be assessed in all patients. However, it shows that a pCR in pancreatic cancer is still associated with a high risk of recurrence when mutations or molecular markers are found in the remaining fibrotic tissues, cells, or in the circulation.

A common pitfall in many studies is that the variation in extent of tissue sampling is not specified even though it is key to compensating for potential intratumoral heterogeneity. Heterogeneity in the tumour might provide an explanation for lack of a molecular response in some instances. Therefore, evaluating the molecular response might give a more precise assessment of tumour response than histopathology, yet it is more comprehensive, costly, and labour intensive. Furthermore, classical and basal-like morphological subtypes seem to have distinct prognostic profiles^8^, suggesting that these subtypes should be reported as part of the evaluation. Furthermore, some have suggested that a high level of GATA6 expression indicates sensitivity to chemotherapy, and hence can be a predictive marker obtainable from biopsy or tissue. Response prediction may even become more important as treatment moves towards total neoadjuvant therapy for some patients.

Studies using novel spatial imaging techniques have reported a remodelling of the tumour immune microenvironment after neoadjuvant therapy towards a more immunogenic state, with higher CD8/CD4 ratios, increase in M1 macrophage phenotype, and decrease in immunosuppressive granulocyte density^9,10^. Incorporating scoring of immune microenvironment components might provide future avenues for tumour response evaluation and potentially open possibilities for immunotherapy. Unfortunately, pancreatic cancer is an immune ‘cold’ tumour, but better understanding of the local tumour immune microenvironment may eventually provide an opportunity to turn some of these cancers into immune ‘hot’ tumours that respond to immunotherapy.

Novel and robust modes of neoadjuvant response evaluation are urgently needed in pancreatic cancer (*Fig. 1b*). The emergence of patient-derived organoids (PDOs), obtained through pretreatment biopsies or from resected tumour specimens, may be the best way forward. Indeed, ability to establish PDOs from chemotherapy-naive and postneoadjuvant therapy tissue enables longitudinal PDO generation to assess dynamic chemotherapy sensitivity profiling^11^. Although establishing PDOs is reported to be successful in up to three-quarters of patients currently, improved technology and turnaround times for *in vivo* drug evaluation may prove the most reliable and tailored response evaluation for future patients.
